# Effects of nitrogen and phosphorus fertilization on soil respiration and its temperature sensitivity in grassland alfalfa soil in Northwest China

**DOI:** 10.3389/fpls.2026.1795072

**Published:** 2026-05-20

**Authors:** Wei Hu, Ahmed H. El-Sappah, Yu Chen, Mohamed F. Abo El-Maati, Yahong Zhang

**Affiliations:** 1 Faculty of Agriculture, Forestry and Food Engineering, Yibin University, Yibin, China; 2 Genetics Department, Faculty of Agriculture, Zagazig University, Zagazig, Egypt; 3 Solid-state Fermentation Resource Utilization Key Laboratory of Sichuan Province, Yibin University, Yibin, China; 4 Sichuan Oil Cinnamon Engineering Technology Research Center, Yibin University, Yibin, China; 5 Department of Biochemistry, Faculty of Agriculture, Zagazig University, Zagazig, Egypt; 6 School of Agriculture, Ningxia University, Yinchuan, China

**Keywords:** forage yield, microbial biomass carbon, *Q*_10_, root mass, soil respiration

## Abstract

**Introduction:**

Understanding the temperature sensitivity (*Q*_10_) of soil respiration (RS) and its response to nitrogen (N) and phosphorus (P) fertilization is essential for predicting soil carbon dynamics under climate change. However, seasonal responses of RS and *Q*_10_ to fertilization in semiarid grasslands remain insufficiently documented.

**Methods:**

A two-year field experiment was conducted from April 2017 to March 2019 in an alfalfa grassland in Northwest China. Four treatments were established: control, N fertilization, P fertilization, and combined NP fertilization. Soil respiration, soil temperature and moisture, forage yield, root biomass, and key soil properties were measured during both growing and non-growing seasons. The temperature sensitivity of soil respiration was calculated using the *Q*_10_ coefficient.

**Results:**

Nitrogen, phosphorus, and NP fertilization significantly increased soil respiration during the growing season, but had no significant effect during the non-growing season. Both N and P applied individually enhanced root biomass throughout the growing season and across the entire year, whereas no significant N × P interaction was detected. *Q*_10_ values were substantially higher during the non-growing season, ranging from 4.91 to 5.54 (mean 5.16), compared with 1.38 to 1.95 (mean 1.68) during the growing season. Fertilization increased *Q*_10_ in both seasons, indicating greater temperature sensitivity of soil carbon release. Soil respiration increased exponentially with soil temperature at 10 cm depth and was positively associated with soil moisture, forage yield, root biomass, total phosphorus, microbial biomass carbon, and soil organic matter, but negatively correlated with soil pH.

**Discussion:**

Nitrogen and phosphorus fertilization altered seasonal patterns of soil respiration and increased the temperature sensitivity of soil carbon emissions. These findings demonstrate that nutrient management can significantly influence carbon cycling and climate feedbacks in dry and semiarid grassland ecosystems.

## 1 Introduction

Soil serves as a significant global reservoir of terrestrial carbon (C), housing an extensive carbon pool (up to 1 meter in depth) comprising 2500 gigatons (1 gigaton = 10^9 tons) of carbon in both organic and inorganic forms. The soil carbon pool exceeds the atmospheric carbon pool by more than threefold (Datta et al., 2015; Lal, 2004; Wani and Qaisar, 2014). Consequently, slight alterations in the soil carbon reservoir would substantially influence future atmospheric carbon dioxide (CO_2_) levels and the current state of climate change (Schlesinger and Andrews, 2000; Smith et al., 2008). Soil respiration (RS) is considered a significant carbon outflow from terrestrial ecosystems to the atmosphere and is crucial in controlling the ecosystem carbon cycle and carbon-climate feedback (Jiang et al., 2015; Wang et al., 2018b). Acquiring a greater understanding of the elements that influence RS is hence essential. Multiple studies indicate that many environmental conditions and ecological processes affect the RS (Stiles et al., 2018). The availability of soil substrates, bulk density, pH, soil temperature, and wetness significantly influence respiration rates by affecting soil microbial activity, root function, and gas diffusion through soil pores (Ganjurjav et al., 2014; Luo et al., 2016; Stiles et al., 2018). The components of vegetation exerted an indirect influence on the RS by altering the net carbon intake from plants, affecting plant degradation resistance, and impacting soil microclimate and structure (Fu et al., 2013; Stiles et al., 2018). The temperature sensitivity of the RS is a significant problem in global warming research (Espíndola et al., 2018; Han et al., 2014; Pang et al., 2013).

The temperature sensitivity of RS is frequently represented by the *Q*_10_ value, which serves as a significant parameter to elucidate the potential interactions between the climate system and the carbon cycle in terrestrial ecosystems (Han et al., 2014; Wang et al., 2016). Soil respiration temperature sensitivity (*Q*_10_) is a key parameter in evaluating soil carbon cycling and its response to global change. Numerous prior pertinent scientific studies have concentrated on RS reactions to soil temperature as a critical variable. Thus, the exponential correlation method has been effectively formulated for application and simulation of *Q*_10_ (Chen et al., 2010; Han and Jin, 2018). The *Q*_10_ value based formula is typically employed to execute the necessary calculations between RS and temperature across both domestic and international scales (Luo et al., 2016; Zhang et al., 2007). The *Q*_10_ value is typically anticipated to resemble that of a year when formulating the annual gaseous carbon budgets of soil. Measurements indicate that the *Q*_10_ value of the RS rate fluctuates annually (Han and Jin, 2018; Meyer et al., 2018; Noh et al., 2017), while the RS response to temperature is contingent upon the seasonality of plant-related biotic and soil-related environmental factors (Feng et al., 2018; Luo et al., 2016). The *Q*_10_ value often decreases as temperature rises. Conversely, the *Q*_10_ generally diminishes as soil moisture decreases (Janssens and Pilegaard, 2003; Qi and Xu, 2001). Both are essential elements influencing microbial growth, community structure, and activity in soil (Luo et al., 2016; Wang et al., 2018b). Likewise, the *Q*_10_ value may be affected by the amount and accessibility of substrates (Gershenson et al., 2009; Wang et al., 2016). Substrates can induce inter-annual fluctuations in *Q*_10_ values, likely due to differences in carbon fixation via plant photosynthesis and belowground carbon allocation (Wan and Luo, 2003; Wagai et al., 2013). Fertilization modifies soil nutrient availability, alters microbial community structure, affects plant growth and root activity, and thereby mediates the temperature sensitivity of soil carbon mineralization (Ding et al., 2010; Song et al., 2016; Wang et al., 2016). Most existing studies have explored *Q*_10_ responses to fertilization during the growing season, yet investigations into its variation during the non-growing season remain scarce. Understanding how fertilization mediates *Q*_10_ variation is thus essential for precisely evaluating soil carbon emission processes and forecasting how nutrient enrichment affects regional carbon balance in managed grassland ecosystems.

Nitrogen (N) and phosphorus (P) are critical nutrients in agricultural soil that can influence vegetation development in terrestrial ecosystems, either favorably or adversely (Manzoni et al., 2010). The application of N and P in agricultural soils might alter physiological and biochemical processes, particularly in ecosystems with significant nutrient deficiencies (Liu et al., 2019; Wang et al., 2016). Soil respiration comprises two key components: autotrophic respiration derived from plant roots and heterotrophic respiration from microbial decomposition. Root-associated processes are particularly sensitive to nutrient availability and environmental conditions, and numerous studies over the past two decades have shown that the effects of nitrogen and phosphorus fertilization on soil microbial metabolism and root respiration can be highly complex (Jiang et al., 2015; Stiles et al., 2018). N fertilization resulted in varying effects on the RS, including stimulation (Fang et al., 2017; Jia et al., 2012), inhibition (Bowden et al., 2004; Wei et al., 2018), or minimal impact (Lee and Jose, 2003). This may be attributed to variations in N fertilization rates, types of vegetation, and monitoring techniques (Fu et al., 2013; Liu et al., 2019). N fertilization has been shown to affect RS variably throughout distinct temporal phases, even within identical vegetation types (Ren et al., 2016). In contrast to the extensive research on the impact of N fertilization on root systems, the comprehension of how augmented P fertilization influences root systems is constrained. Recently, conflicting conclusions have emerged regarding the effects of P fertilization on RS. For instance, specific research has indicated that P fertilization has beneficial impacts on root systems (Cleveland et al., 2002; Liu et al., 2012). Additional research revealed that P fertilization reduced RS levels (Feng and Zhu, 2019; Song et al., 2011). The N:P ratio in agricultural soil can impact soil microbial biomass carbon (MBC), plant biomass production, photosynthetic rate, and various biochemical activities (Wang et al., 2016). Soil respiration exhibits notable seasonal variations in its response to nitrogen (N) and phosphorus (P) fertilization (Li et al., 2015; Liu et al., 2019). Key factors driving such seasonal differences include shifts in soil temperature and moisture conditions across seasons, variations in root physiological activity, changes in microbial metabolic efficiency, fluctuations in the quantity and quality of soil substrates, as well as distinct plant nutrient requirements between growing and non-growing periods (Fang et al., 2017; Luo et al., 2016). These changes collectively lead to obvious seasonal divergence in soil respiration responses to fertilization. Despite this, most prior research has concentrated solely on the growing season, leaving the response patterns and underlying regulatory mechanisms of soil respiration to N and P fertilization during the non-growing season largely unaddressed (Luo et al., 2016). To fully and accurately clarify how N and P addition affects soil respiration and its temperature sensitivity, a comprehensive full-year study covering both growing and non-growing seasons is therefore indispensable.

Alfalfa (*Medicago sativa* L.) is a significant cash crop and a valuable component of the plant community in the arid regions of Northwest China (Xiao et al., 2015), playing a crucial role in ecological restoration and the optimization of local agropastoral production systems. Given the soil nutrient deficiency in the study area, fertilization is essential to ensure alfalfa growth and maintain ecological stability. Specifically, nitrogen (N) and phosphorus (P) can significantly alter plant growth and soil carbon emission processes by regulating root growth, microbial biomass, and soil hydrothermal conditions (Fan et al., 2016; Li et al., 2018). Nevertheless, the data regarding the responses of RS and *Q*_10_ values to N and P fertilization are scarce and inadequate, particularly regarding the underlying mechanisms. Few studies have examined RS dynamics across both growing and non-growing seasons, despite the substantial contribution of the non-growing season to the annual carbon budget. This study assessed the RS, soil characteristics, and biological markers in alfalfa pasture within the arid region of Northwest China from April 2017 to April 2019. We proposed the following core hypotheses: Nitrogen and phosphorus fertilization significantly affect *Rs* and *Q*_10_, with distinct differences in effects between growing and non-growing seasons (Hypothesis 1). The seasonal variations in the response of Rs to fertilization are mainly regulated by soil abiotic factors (temperature, moisture, nutrient content) and plant growth status(Hypothesis 2). The effect of fertilization on Rs is weaker in the non-growing season due to low temperature and limited plant physiological activity (Hypothesis 3). Guided by these hypotheses, the primary objectives of this study were to examine the dynamics of soil respiration (RS) during the growing and non-growing seasons and the response of its temperature sensitivity to N and P fertilization. Furthermore, evaluate the interactive effects of N and P fertilization on RS and its temperature sensitivity. Finally, explore the regulatory roles of soil abiotic factors and plant growth status in the response of Rs to fertilization.

## 2 Materials and methods

### 2.1 Study area

**Figure 1:**
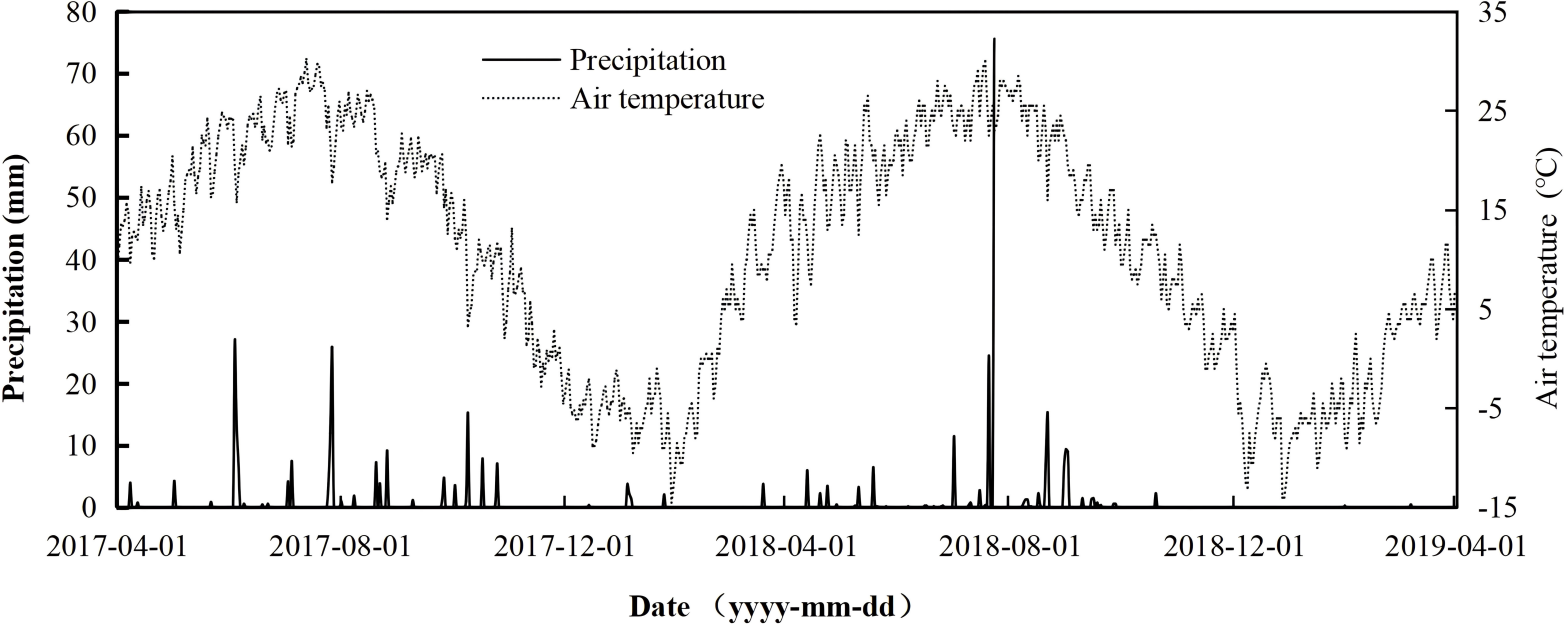
Variation of precipitation (mm) and air temperature (°C) during the experiment period from 2017 to 2019.

The study was conducted between 2017 and 2019 at Maosheng Agricultural Station in Yinchuan, the capital city of Ningxia Hui Autonomous Region, Northwest China (38°31′N, 106°8′E; 1037 m a.s.l.). Irrigation water from the Yellow River has transformed a part of the region. This region has a continental arid, temperate climate, characterized by hot, dry summers and cold winters (Huang et al., 2018). The annual mean precipitation is 203mm, 80% of which occurs between April and October. The mean yearly temperature is about 8.5 °C, and the maximum and minimum mean monthly temperatures are 24.8°C in July and -12.3°C in January, respectively (**Figure 1**). The mean annual pan evaporation is approximately 2250 mm. The annual frost-free period is 157 days, and the mean annual sunshine hours are 3075.5h. The soil at this study site is typically classified as a light sierozem, primarily composed of sand and silt (48.7% and 38.6%), with 12.7% clay (Huang et al., 2018). The physical and chemical properties of the upper soil layer (0–20 cm) were measured at the beginning of the field experiment in 2017. The available soil N, P, and K of this soil type are 43.91 mg kg^-1^, 10.65 mg kg^-1,^ and 128.26 mg kg^-1^, respectively. The concentration of soil organic matter is 13.51 g kg^-1^, and the total soil salt content is 0.26 g kg^-1^. The total N concentration is 0.81 g kg^-1^, and the total P concentration is 0.58 g kg^-1^. Soil bulk density is 1.51g cm^-3^, and soil pH is 8.61. The study area was sown with alfalfa on 16 May 2016 using a grain drill in rows with a row spacing of 22.5 cm and a seed rate of 22.5 kg hm^-2^. The alfalfa (*Medicago sativa*) cultivar is “Magnum VII” (high yield, extraordinary drought tolerance, and cold resistance) from America. Management was similar to the local field practice. The above-ground parts of alfalfa were harvested three times in 2016, and four times in 2017 and 2018. The spring green-up began on 1 April in 2017, 3 April in 2018 and 5 April in 2019, respectively. The autumn senescence occurred on 25 October 2017 and 21 October 2018, respectively.

### 2.2 Experimental design and field management

The experiment was designed as a random block with three triplicate blocks, separated by 2 m walkways. In each block, four plots (4 m × 6 m) were established (4 treatment×3 replicates=12 plots). According to local fertilization practices, no potassium-containing fertilizers were applied at any stage of the experiment. All treatments received only nitrogen and phosphorus fertilizers, with four fertilization treatments included as follows: no fertilization (CK), 120 kg N ha^-1^yr^-1^ (N), 120 kg P ha^−1^ yr^−1^ (P), and 120 kg N ha^−1^ yr^−1^ and 120 kg P ha^−1^ yr^−1^ (NP). N fertilizer (urea, 46.0% N) and P fertilizer (calcium superphosphate, 18% P_2_O_5_) were manually added to the grassland surface at a spacing of 15cm in 2017 and 2018. The annual amount of fertilizer was divided into four applications, with 40%, 30%, 20%, and 10% applied in early April, June, July, and August, respectively, with each irrigation event. The experiment was conducted using the flood irrigation system. According to the local farmer’s long-standing irrigation practices, a total of eight applications, each at 1,200 mm per year, were made in each plot, regardless of the treatment. Weed management and insect control were performed as recommended for the crop when necessary. Four harvests in 2017 occurred sequentially on 29 May, 27 June, 1 August, and 20 September, and four harvests in 2018 occurred sequentially on 5 June, 7 July, 11 August, and 26 September.

### 2.3 Measurements of the RS, soil temperature, and moisture

The RS was measured using a portable infrared gas analyzer attached to a 10 cm diameter closed chamber (EGM-4 and modified SRC-1, PP System, Amesbury, MA, USA). After alfalfa sowing, three PVC collars (10 cm in diameter × 6 cm in height) were randomly inserted into the soil between alfalfa rows in each plot and remained undisturbed for one year to avoid soil disturbance.

The RS was measured three times per month during the growing season (April to October) and twice per month during the non-growing season (November to March of the following year) from April 2017 to March 2019. At least two measurements were taken for each sampling plot, and the average of both measurements was taken as RS. All RS measurements were collected between 09:00 and 11:00 local time on sunny days to minimize sampling deviation caused by daytime (Iqbal et al., 2010). All soil respiration measurements were performed at least three days after rainfall or irrigation events. Before each measurement, the chamber was flushed with ambient air for 15 s and then placed on the pre-inserted PVC collars to isolate the measurement zone from the atmosphere. The sampling period lasted for 120 s, precisely, until the internal CO_2_ concentration in the chamber reached a constant flux rate. These procedures effectively minimized disturbances from atmospheric gas exchange, ensuring the reliability of measured soil respiration fluxes. The RS was then calculated by fitting a quadratic equation to track and study the relationship between the increase in CO_2_ concentration and elapsed time (Mills et al., 2011). Soil temperature (ST) was monitored at 10 cm (depth) by a temperature sensor (ZDR-41, Zeda Instrument Co., Ltd., Hangzhou, Zhejiang, China). The ICT MP406 Digital Soil Moisture Kit (ICT International, Armidale, NSW, Australia) was used to determine the soil water content (SWC) using wave sensors. In this study, the topsoil completely freezes during winter. Soil moisture sensors based on dielectric principles cannot yield reliable data under frozen conditions, because the dielectric properties of ice differ significantly from those of liquid water. Furthermore, repeated freeze-thaw cycles may damage the sensor probes. Accordingly, soil moisture sensors were removed before soil freezing, and no soil moisture data were collected during the winter period.

### 2.4 Forage yield and root biomass

Forage yield was manually harvested in the early flowering stage with a stubble height of 5 cm, when 10% of the alfalfa had bloomed. Four forage harvests were performed every year. At each harvest, three squares (1m×1m) of fresh alfalfa were selected in the diagonals of each plot. Samples were dried at 105 °C for 15 min and then kept at 65 °C until a constant weight was achieved to determine the forage yield. The root biomass was collected after the fourth harvest was completed (Xiao et al., 2015). Collection of root biomass samples was performed by digging a soil profile (0.5 cm × 0.5 m × 0.6 m), with three parallel profiles in each plot (Huang et al., 2018). Root biomass samples from the 0-60cm soil layer were taken at 10cm intervals using a spade, and the sample volumes were 30 cm×30 cm×10 cm (covered one row of alfalfa). The roots were separated from the soil by rinsing and then brought back to the laboratory. They were oven-dried at 105 °C for 1 h and subsequently at 65 °C to a constant mass, after which they were weighed to estimate root biomass (Li et al., 2018).

### 2.5 Soil sampling and analysis

Soil samples were collected after the fourth harvest of alfalfa in October 2017 and 2018, respectively. Six soil cores (5 cm diameter) of each subplot were randomly taken from the top layer (0-20 cm) using a soil drill and then mixed into a composite sample. The samples were passed through a 2 mm sieve to remove roots and stones, and divided into two parts. One part of fresh soil was used for the analysis of microbial biomass carbon and soil organic matter. The other part was air-dried for the determination of soil pH, the available N and P concentrations, and the total N and P concentrations. Soil microbial biomass carbon (MBC) was determined using the chloroform-fumigation extraction method (Chen et al., 2019). Soil organic matter (SOM) was determined using potassium dichromate oxidation titration. Soil pH was assessed using a glass electrode in a 1:2.5 (w/v) soil-to-water solution. The available N (AN) and available P (AP) of the soil sample were measured using the procedures described by Gong et al. (2015). The total N (TN) was measured by the semi-micro Kjeldahl method. The total P (TP) was measured using the molybdenum antimony anti-colorimetric method (Xia et al., 2013).

### 2.6 Date analysis

An exponential equation was applied to represent the relationship between the RS rate and ST (Fang et al., 2017; Sun et al., 2018). The temperature sensitivity coefficient (b) of soil respiration was calculated according to Equation 1:

(1) $$RS=\text{a}e^{bT}$$

*where:* RS (g CO_2_·m^-2^ h^-1^) refers to the measured RS rate, T (°C) refers to ST at 10 cm (depth), a refers to the intercept of the RS at ST of 0 °C, and b refers to the coefficient of temperature sensitivity of the RS.

The temperature sensitivity of the RS (*Q*_10_) was calculated using the following (Sun et al., 2018; Wang et al., 2016). Q10 was calculated according to Equation 2:

(2) $$Q_{10}=\text{ e}10b$$

The growing-season, non-growing-season, and annual cumulative RS were calculated as the sum of RS rates of each seasonal measurement period of the year (Ding et al., 2010; Feng et al., 2018). Cumulative soil respiration (CSR) was calculated according to Equation 3:

(3) $$\text{CSR}=\text{Σ}\left(\text{Δtk }\times \text{ Fk}\right)$$

Where CSR(g CO_2_-C·m^-2^) is cumulative RS, Δtk(=tk−tk_−1_) is the time interval between each field measurement within the season, and Fk is the average RS rates over the interval tk−tk_−1_.

The effects of experimental treatments on the following factors were assessed: soil chemical variables and temperature, forage yield, root biomass, RS, and *Q*_10_. These effects were evaluated using a multi-way ANOVA variance analysis and Fisher’s LSD test for comparisons of means. In all tests, a significance level of P = 0.05 was used. The repeated-measure ANOVA was used to test the effects of N fertilization, P fertilization, and the year on the seasonal RS, and the interactions between them. Additionally, the linear regression analysis was conducted between RS and soil moisture, forage yield, and root biomass. Relationships between RS and these parameters were analyzed using Pearson correlations and regression analysis. All statistical analyses of the data were conducted using the SPSS software package for Windows (SPSS 13.0, SPSS Inc., Chicago, IL, USA).

## 3 Results

### 3.1 Environmental conditions and soil properties

**Figure 2:**
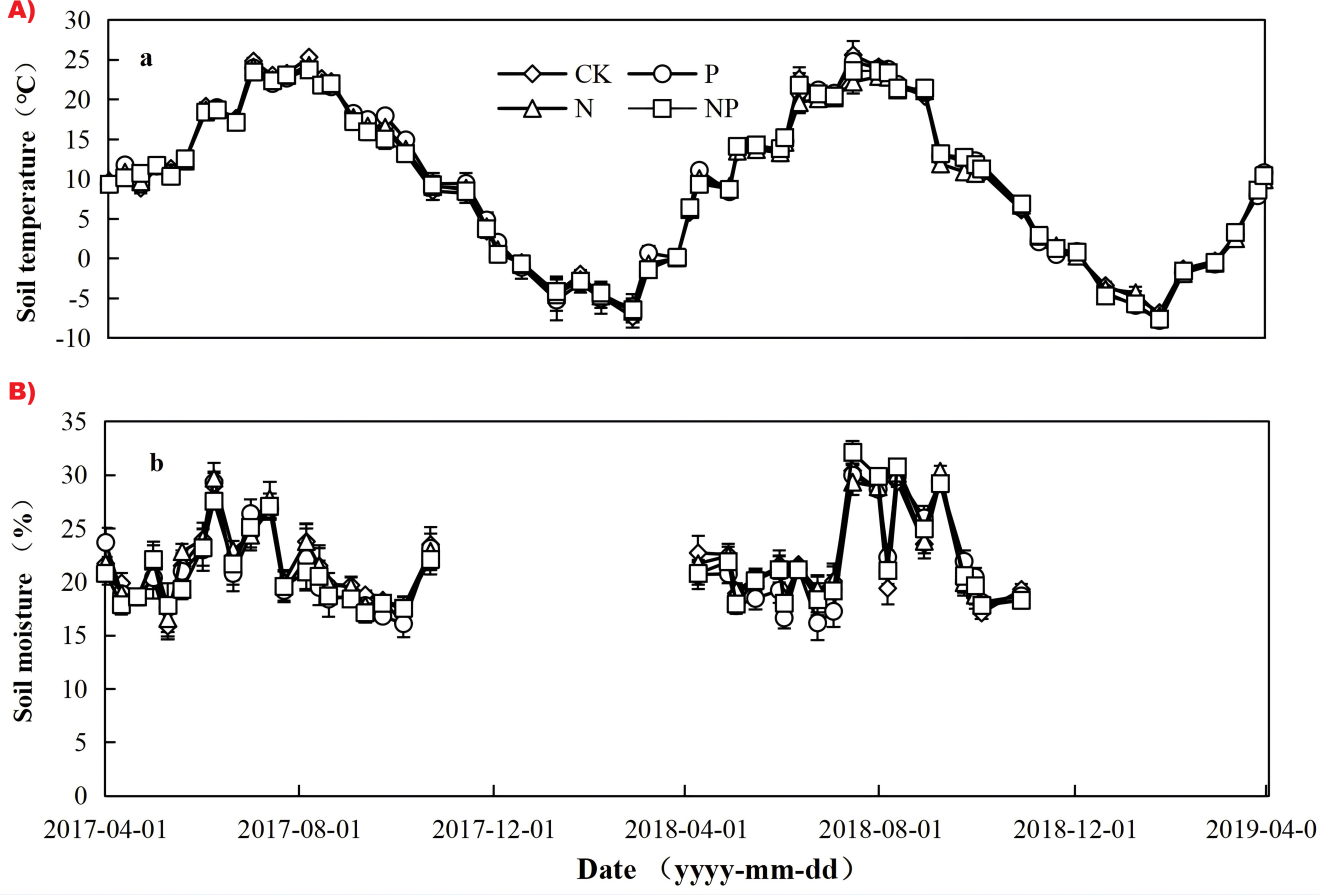
Variation in soil microclimatic conditions during the experimental period (2017 to 2019). **(a)** Soil temperature (°C). **(b)** Soil moisture (%). Data are shown for CK (control), P (phosphorus addition), N (nitrogen addition), and NP (combined nitrogen and phosphorus addition). Values represent mean ± standard error.

Over the two years, ST at 10 cm (depth) for CK, N, P, and NP treatments showed obvious seasonal patterns (**Figure 2a**), which were in explicit agreement with the variation in air temperature (**Figure 1**). ST peaked on 5 August in 2017 and 14 July in 2018. The mean temperature of ST over the study period was 10.6 °C, 10.7 °C, 10.4 °C, and 10.5 °C in the CK, N, P, and NP treatments, respectively. In contrast, no seasonal pattern of soil moisture was observed throughout the study period (**Figure 2b**). The mean soil moisture at 0–10 cm (depth) during the growing season over the study period was 21.7%, 21.3%, 21.9% and 21.5% in CK, N, P, and NP treatments, respectively.

During the measurement period, the soil pH under N, P, and NP treatments decreased significantly by 0.16, 0.22, and 0.26 units in 2017, and by 0.15, 0.21, and 0.22 units in 2018, compared with the control (**Table 1**). N fertilization alone significantly increased soil AN and SOM, but did not substantially affect TN and AP (**Table 1**). P fertilization alone significantly augmented AP and TP. The NP treatment increased TN and TP in 2017 and 2018, while no significant differences in total N content were observed among the CK, P, N, and NP treatments (**Table 1**). Furthermore, soil MBC under N, P, and NP treatments increased by 4.3%, 14.6%, and 15.7% in 2017, and by 1.6%, 11.6%, and 8.6% in 2018, respectively (**Table 1**).

**Table 1:** Basic physicochemical characteristics of soil (0–20 cm) under different treatments in 2017 and 2018. .

Year	Treatments	pH	TN(g kg^-1^)	TP(g kg^-1^)	AN(/mg kg^-1^)	AP(mg kg^-1^)	MBC(mg kg^-1^)	SOM(g kg^-1^)
2017	CK	8.55 ± 0.09a	0.91 ± 0.06a	0.67 ± 0.03b	61.15 ± 3.39b	11.24 ± 1.29c	783.32 ± 11.53b	15.97 ± 0.52b
	N	8.39 ± 0.07b	0.94 ± 0.09a	0.70 ± 0.02b	71.37 ± 7.81a	10.36 ± 1.33c	816.65 ± 11.87b	18.06 ± 0.49a
	P	8.33 ± 0.03b	0.99 ± 0.08a	0.81 ± 0.05a	63.91 ± 4.89b	14.01 ± 1.14b	897.93 ± 10.96a	16.87 ± 0.96ab
	NP	8.29 ± 0.04b	1.04 ± 0.07a	0.86 ± 0.04a	74.72 ± 6.63a	15.16 ± 1.41a	906.55 ± 11.52a	17.22 ± 0.99a
2018	CK	8.48 ± 0.03a	0.98 ± 0.27a	0.71 ± 0.06b	62.86 ± 1.47b	12.79 ± 1.49b	849.99 ± 11.56b	16.97 ± 0.49b
	N	8.33 ± 0.08ab	1.07 ± 0.17a	0.76 ± 0.07b	73.07 ± 2.49a	13.36 ± 1.13b	863.96 ± 16.17ab	18.34 ± 0.83a
	P	8.27 ± 0.09b	0.92 ± 0.24a	0.94 ± 0.06a	67.73 ± 4.96b	15.98 ± 1.65a	948.92 ± 19.78a	17.69 ± 0.76ab
	NP	8.26 ± 0.12b	1.03 ± 0.25a	0.97 ± 0.09a	70.23 ± 2.64a	16.33 ± 1.37a	923.21 ± 16.44ab	18.46 ± 0.86a

Different lowercase letters indicate statistically significant differences among treatments within the same year at the P<0.05 level.

### 3.2 Variation in the RS

**Figure 3:**
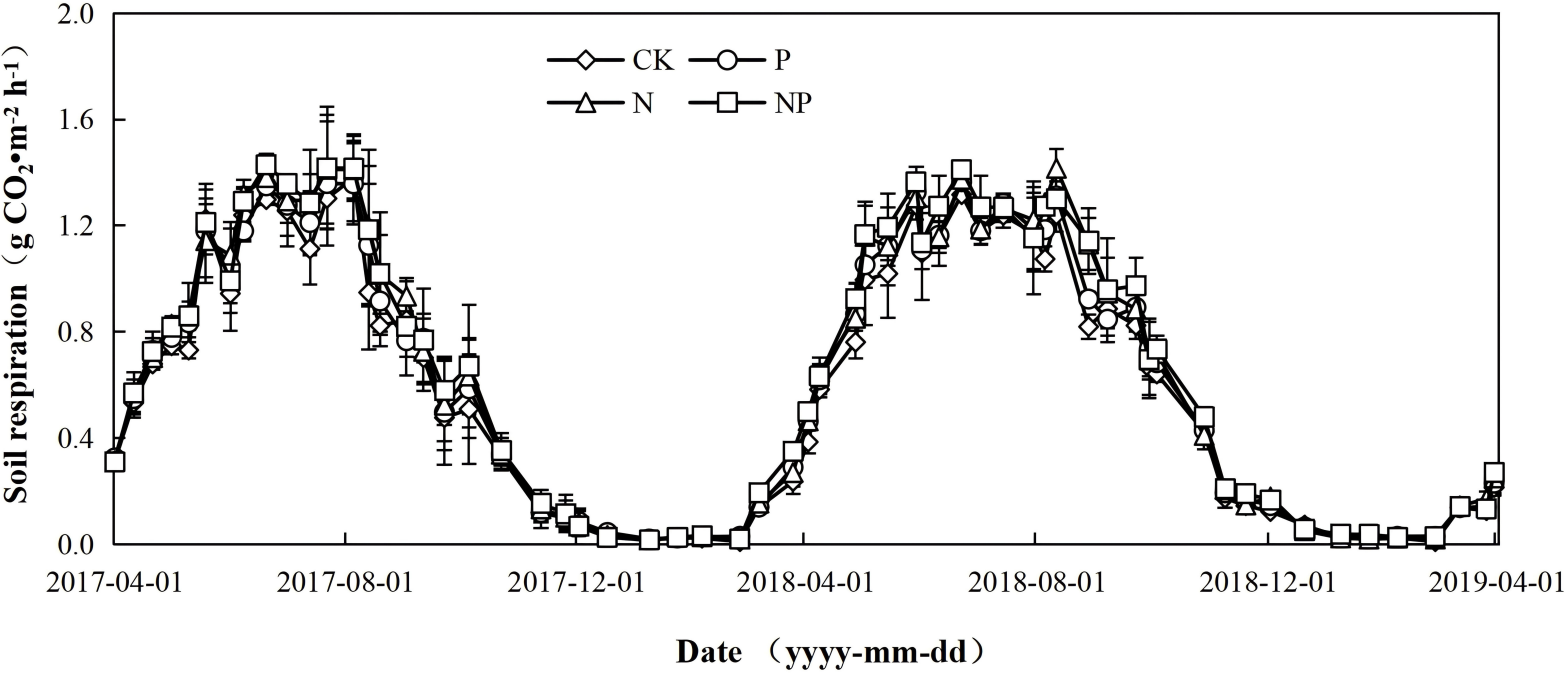
Dynamics of the RS(g CO_2_·m^-2^ h^-1^)from 2017 to 2019 in an alfalfa grassland under different treatments.

The RS showed similar seasonal and annual variances as ST in the CK, N, P, and NP treatments of the two years, which were consistently lower in the winter season, then increased rapidly in April and reached a maximum between July and August (**Figures 2a**, **3**). Compared with the control, N fertilization alone significantly stimulated RS during the growing season by 9.2% in 2017 and 8.5% in 2018 (**Table 2**). In 2017 and 2018, P fertilization alone enhanced RS during the growing season by 4.6% and 5.3%, respectively (**Table 2**). P fertilization alone significantly increased RS during the growing season in 2018, but did not substantially affect RS during the growing season in 2017 (**Table 2**). The NP treatment significantly increased RS during the growing season by 10.3% in 2017 and 10.6% in 2018 (**Table 2**). However, there were no differences in the RS rate among the CK, N, P, and NP treatments in the two years during the non-growing season (**Table 2**). The repeated-measure ANOVA test of the RS showed that N fertilization or P fertilization had a significant effect (*P*<0.05) on RS during the growing season and the whole year, while no interactive effects of N and P fertilization were observed. Furthermore, neither N fertilization nor P fertilization showed significant interactions with year (**Table 3**). Moreover, the mean whole year RS rate (calculated by averaging two whole years) in the N treatment (0.68 g CO_2_·m^-2^ h^-1^), P treatment (0.66 g CO_2_·m^-2^ h^-1^) and the NP treatment (0.69 g CO_2_·m^-2^ h^-1^) was 7.9%, 4.8% and 9.5% higher than that in the CK treatment (0.63 g CO_2_·m^-2^ h^-1^) (**Table 2**).

**Table 2:** Mean RS(g CO_2_·m^-2^ h^-1^)under nitrogen and phosphorus treatments from 2017 to 2019.

Treatments	2017-2018			2018-2019			Mean		
	Growing	Non-growing	Whole year	Growing	Non-growing	Whole year	Growing	Non-growing	Whole year
CK	0.87 ± 0.09b	0.08 ± 0.01a	0.61 ± 0.06b	0.94 ± 0.05c	0.11 ± 0.01a	0.64 ± 0.03b	0.90 ± 0.05b	0.09 ± 0.01a	0.62 ± 0.02b
N	0.95 ± 0.07a	0.09 ± 0.01a	0.66 ± 0.05a	1.02 ± 0.03a	0.12 ± 0.01a	0.70 ± 0.05a	0.98 ± 0.03a	0.10 ± 0.02a	0.68 ± 0.02a
P	0.91 ± 0.07ab	0.09 ± 0.01a	0.64 ± 0.05ab	0.99 ± 0.03b	0.11 ± 0.01a	0.68 ± 0.03ab	0.95 ± 0.04a	0.10 ± 0.01a	0.66 ± 0.04ab
NP	0.96 ± 0.09a	0.10 ± 0.01a	0.67 ± 0.06a	1.04 ± 0.06a	0.12 ± 0.02a	0.71 ± 0.02a	1.00 ± 0.05a	0.11 ± 0.01a	0.69 ± 0.03a

2017-2018 represents the period from March 28, 2017 to April 3, 2018; 2018-2019 represents the period from April 3, 2018 to April 5, 2019; Mean indicates the average value from March 28, 2017 to April 5, 2019. Different lowercase letters indicate significant differences among treatments at *P* < 0.05, while the same letters indicate no significant difference at *P*>0.05. values are means of triplicate ± SE, the same below.

**Table 3:** Effects of factors on the RS (g CO_2_·m^-2^ h^-1^) of the Year, N, P fertilizations, and their interaction were tested by repeated-measure ANOVA during the experiment period from 2017 to 2019.

Source of variation	Degrees of freedom	Growing		Non-growing		Whole year	
		*F*-Value	*P*	*F*-Value	*P*	*F*-Value	*P*
Block	2	1.05	0.04	1.04	0.38	1.22	0.03
Year	1	12.33	<0.01	29.19	<0.001	6.19	0.03
N	1	1.97	0.018	3.72	0.07	2.23	0.015
P	1	9.74	<0.01	3.97	0.08	9.25	<0.01
Year×N	1	0.09	0.76	0.59	0.45	0.08	0.78
Year×P	1	0.02	0.89	0.003	1.01	0.003	0.99
N×P	1	0.49	0.49	0.15	0.71	0.52	0.48
Year×N×P	1	0.006	0.93	0.001	0.99	0.003	0.96

### 3.3 Cumulative the RS

**Figure 4:**
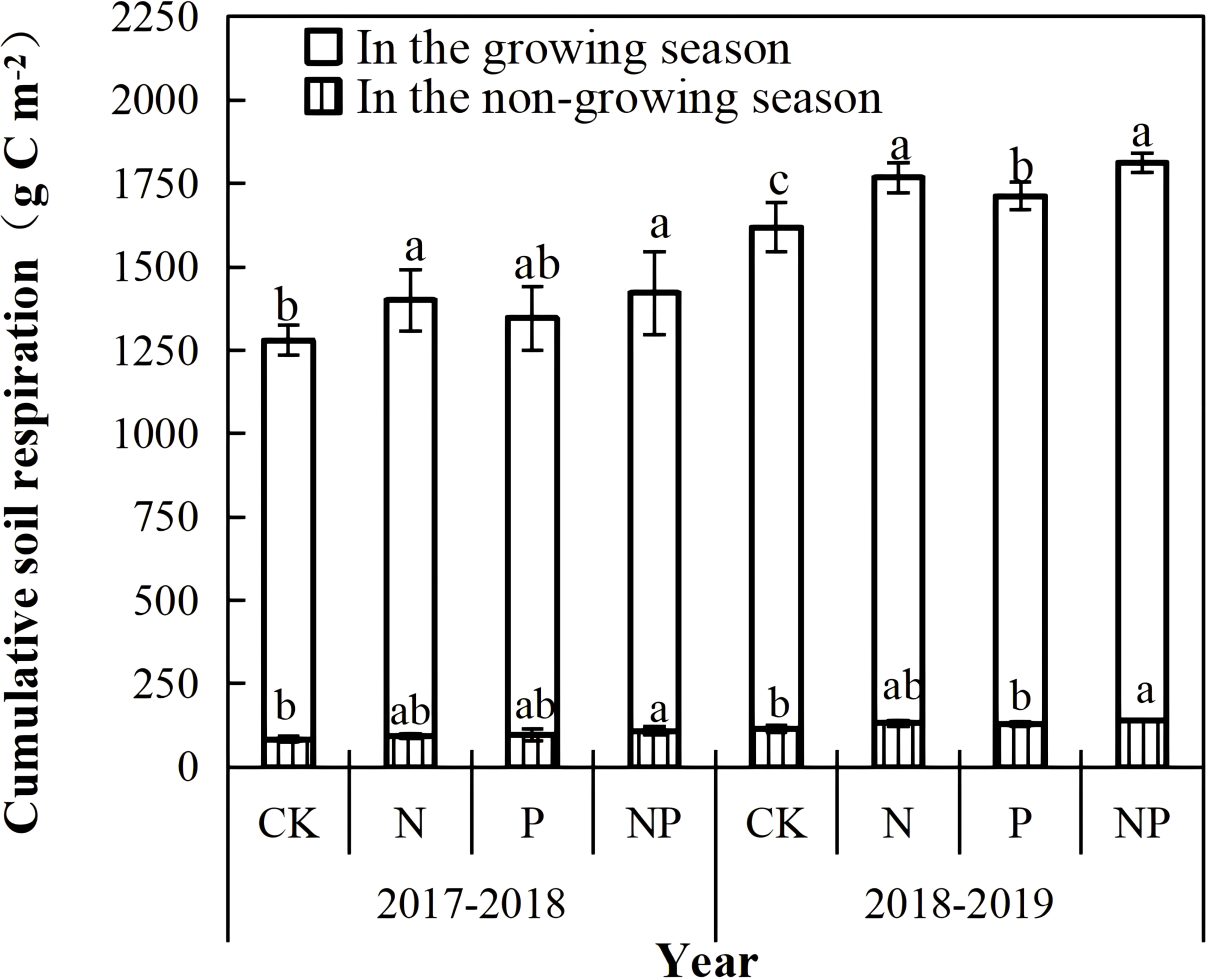
Cumulative respiration (g C m^-^²) during the growing season and non-growing season under different nitrogen and phosphorus treatments from 2017 to 2019. Different lowercase letters indicate significant difference among treatments within the same year (P<0.05)

During the four treatments studied, the cumulative RS in the growing season was significantly higher than that in the non-growing season (**Figure 4**). N fertilization alone significantly stimulated the cumulative RS in the ever-increasing season by 9.2% in 2017 and 8.7% in 2018, compared with that of the control, while it did not significantly influence the cumulative RS in the non-growing season over the two years. P fertilization alone did not significantly influence the cumulative RS in the 2017 growing season, but it increased it by 5.3% in 2018. However, P fertilization alone did not significantly influence the cumulative RS in the non-growing season in 2017-2018 and 2018-2019. Cumulatively, the RS in either growing season or non-growing season was significantly higher in the NP treatment than in the other treatments. In addition, there was a significant difference in either growing season or non-growing season between the P and NP treatments in 2017-2018, but no significant differences among the N and NP treatments in 2017-2018 and 2018-2019 were observed.

### 3.4 Temperature sensitivity of the RS

During the measurement period, the RS increased exponentially with ST at 10 cm (depth) during the growing season, non-growing season, and the whole year over the two years (**Table 4**). R^2^ values indicated that the effects of ST accounted for 48%–65% of the variance in RS during the growing season, 51%–66% during the non-growing season, and 75%–84% over the two years. The *Q*_10_ value in the non-growing season was significantly higher than that in the growing season and the whole year (**Table 4**). The *Q*_10_ value in the growing season ranged from 1.78 to 1.95, with a mean of 1.87 in 2017, and from 1.38 to 1.57, with a mean of 1.49 in 2018. The *Q*_10_ value in the non-growing season ranged from 4.91 to 5.31 with a mean of 5.11 in 2017-2018 and from 4.96 to 5.54 with a mean of 5.21 in 2018-2019. The *Q*_10_ value in the whole year ranged from 3.63 to 4.06 with a mean of 3.88 in 2017-2018 and from 3.13 to 4.22 with a mean of 3.61 in 2018-2019. Over the same period, the *Q*_10_ value of the NP treatment was significantly higher than that of the other treatments in 2017-2018, but it was lower than that of the N treatment in 2018-2019.

**Table 4:** Regression equations between RS and ST, and *Q*_10_ values under different nitrogen and phosphorus treatments from 2017 to 2019.

Year	Treatments	Growing season	Non-growing season	Whole year
2017-2018	CK	RS=0.30e^0.058T^, R^2^ = 0.52^**^, *Q*_10_ =1.78 ± 0.06b	RS=0.06e^0.162T^, R^2^ = 0.64^**^, *Q*_10_ =5.03 ± 0.13ab	RS=0.07e^0.129T^, R^2^ = 0.82^**^, *Q*_10_ =3.63 ± 0.17b
	N	RS=0.29e^0.065T^,R^2^ = 0.57^**^, *Q*_10_ =1.92 ± 0.04a	RS=0.07e^0.159T^, R^2^ = 0.61^**^, *Q*_10_ =4.91 ± 0.21b	RS=0.07e^0.138T^, R^2^ = 0.84^**^, *Q*_10_ =4.01 ± 0.13a
	P	RS=0.31e^0.061T^, R^2^ = 0.48^**^, *Q*_10_ =1.83 ± 0.11a	RS=0.07e^0.164T^, R^2^ = 0.62^**^, *Q*_10_ =5.17 ± 0.15ab	RS=0.08e^0.133T^, R^2^ = 0.83^**^, *Q*_10_ =3.81 ± 0.13b
	NP	RS=0.29e^0.067T^, R^2^ = 0.59 ^**^, *Q*_10_ =1.95 ± 0.06a	RS=0.07e^0.167T^, R^2^ = 0.54^**^, *Q*_10_ =5.31 ± 0.18a	RS=0.08e^0.141T^, R^2^ = 0.82^**^, *Q*_10_ =4.06 ± 0.14a
2018-2019	CK	RS=0.46e^0.032T^, R^2^ = 0.61 ^**^, *Q*_10_ =1.38 ± 0.06b	RS=0.06e^0.161T^, R^2^ = 0.51^**^, *Q*_10_ =4.96 ± 0.26b	RS=0.08e^0.114T^,R^2^ = 0.75^**^, *Q*_10_ =3.13 ± 0.05c
	N	RS=0.49e^0.045T^, R^2^ = 0.65 ^**^, *Q*_10_ =1.57 ± 0.57a	RS=0.07e^0.171T^, R^2^ = 0.66^**^, *Q*_10_ =5.54 ± 0.28a	RS=0.09e^0.144T^, R^2^ = 0.81^**^, *Q*_10_ =4.22 ± 0.17a
	P	RS=0.516e^0.039T^, R^2^ = 0.58^**^, *Q*_10_ =1.48 ± 0.06ab	RS=0.083e^0.163T^,R^2^ = 0.63^**^, *Q*_10_ =5.10 ± 0.27ab	RS=0.108e^0.125T^,R^2^ = 0.81^**^, *Q*_10_ =3.51 ± 0.15b
	NP	RS=0.53e^0.041T^, R^2^ = 0.60^**^, *Q*_10_ =1.51 ± 0.08ab	RS=0.08e^0.165T^, R^2^ = 0.60^**^, *Q*_10_ =5.25 ± 0.15ab	RS=0.11e^0.127T^,R^2^ = 0.80^**^, *Q*_10_ =3.57 ± 0.13b

* indicates *P* < 0.05, ** indicates *P* < 0.01; the same is shown below.

Different lowercase letters indicate statistically significant differences among treatments within the same year at the p<0.05 level.

### 3.5 Effects of N and P fertilization on the forage yield and root biomass

Forage yields under CK, N, P, and NP treatments were 1.36, 1.64, 1.54, and 1.81 kg m^-2^ in 2017, 1.82, 2.11, 2.24, and 2.32 kg m^-2^ in 2018, respectively (**Table 5**). N and P treatments significantly increased forage yield in 2017 and 2018 compared to the control, with no significant difference between the two treatments (**Table 5**). N fertilization alone significantly stimulated root biomass by 22.4% in 2017 and 16.4% in 2018, compared to that of the control. P fertilization alone significantly increased the root biomass by 35.8% in 2017 and 24.1% in 2018 (**Table 5**). The NP treatment yielded higher forage yields and root biomass than the other treatments in both 2017 and 2018. However, there were no significant differences in root biomass in 2017 and forage yield in 2018 among P and NP treatments.

**Table 5:** Mean forage yield (kg m^-2^) and root biomass (kg m^-2^) under different treatments from 2017 to 2019.

Treatments	2017		2018		Mean	
	Forage yield	Root biomass	Forage yield	Root biomass	Forage yield	Root biomass
CK	1.36 ± 0.11c	0.67 ± 0.07c	1.82 ± 0.14c	0.79 ± 0.05c	1.59 ± 0.17c	0.73 ± 0.04c
N	1.64 ± 0.15ab	0.82 ± 0.06b	2.11 ± 0.16b	0.92 ± 0.07b	1.87 ± 0.19b	0.87 ± 0.05b
P	1.54 ± 0.19b	0.91 ± 0.08ab	2.24 ± 0.19ab	0.98 ± 0.07b	1.89 ± 1.12b	0.95 ± 0.06ab
NP	1.81 ± 0.14a	0.95 ± 0.04a	2.32 ± 0.21a	1.11 ± 0.09a	2.07 ± 1.22a	1.03 ± 0.07a

Different lowercase letters indicate statistically significant differences among treatments within the same year at the p<0.05 level.

### 3.6 Relationship between the RS and biotic and abiotic factors

**Figure 5:**
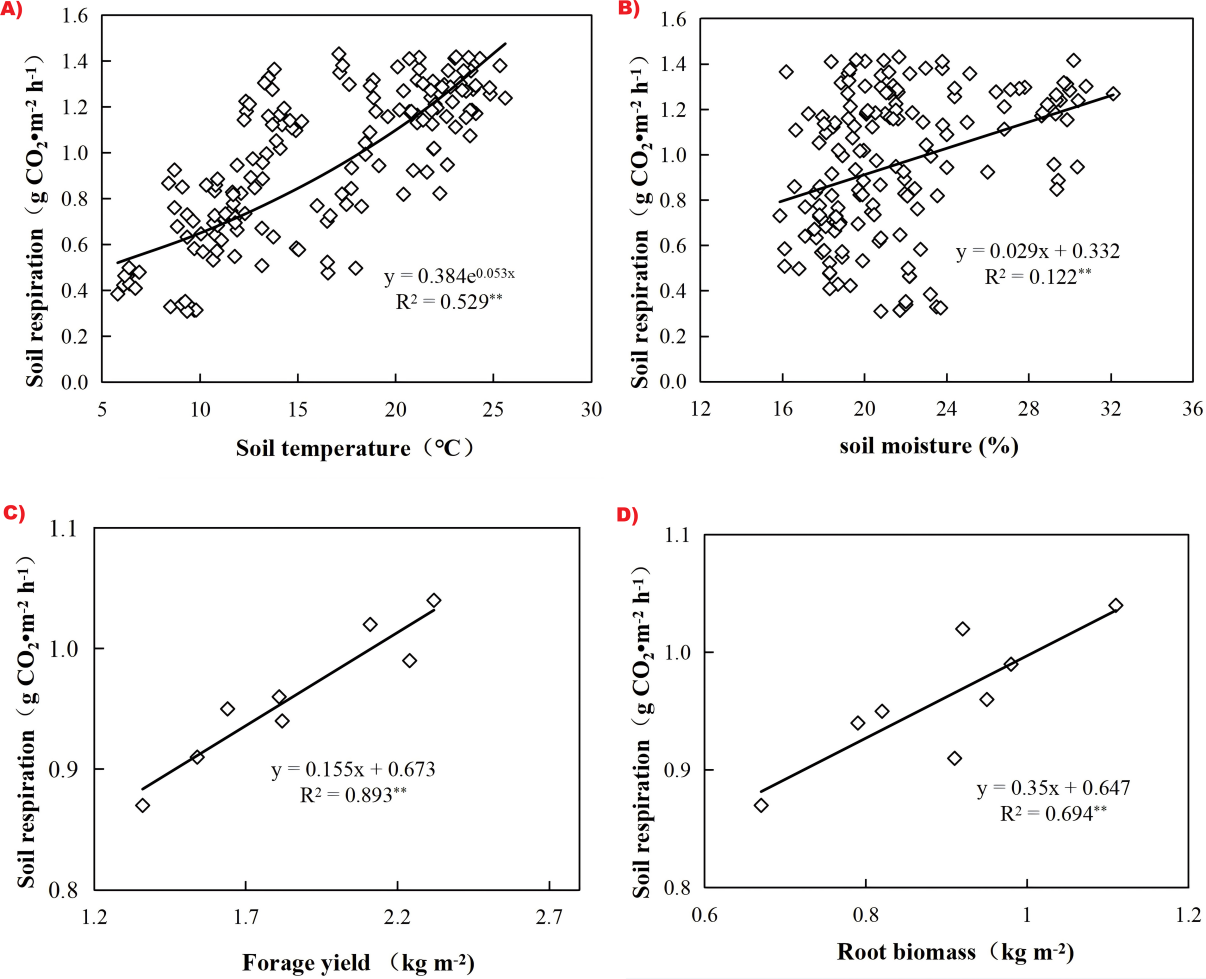
Dependence of the RS(g CO_2_·m^-2^ h^-1^)on ST (°C), soil moisture (%), forage yield (kg m^-2^), and root biomass (kg m^-2^) in the growing season across the two years. Each point represents each observation during the two years under the four different treatments **(a, b)**, and the average value of the RS in the growing season and forage yield and root mass under the same treatment **(c, d)**.

The observations collected for all treatments in the growing season over the study period confirmed that RS extremely significantly increased at 10-cm in depth (R^2^ = 0.529, *P*<0.01) as ST increased, and linearly increased with soil moisture, forage yield, and root biomass as shown in **Figure 5** (R^2^ = 0.122, 0.893, and 0.694, *P*<0.01), respectively. ST and soil moisture could explain 52.9% and 12.2% of the RS variation during the growing season (**Figures 5a, b**). Furthermore, RS increased with increasing forage yield and root biomass in the growing season, to some extent (**Figures 5c, d**). As shown in **Table 6**, RS had a significant positive correlation with total P (TP), microbial biomass carbon (MBC), and soil organic matter (SOM), but a highly significant negative correlation with pH. In addition, there was no significant correlation between RS and the total N (TN), available P (AP), or available N (AN) (*P* > 0.05).

**Table 6:** Pearson’s correlation between the RS and climate factors, soil physicochemical characteristics, and forage yield and root biomass.

Indicators	RS	T	SWC	pH	TN	TP	AN	AP	MBC	SOM	RM	FY
RS	1.000											
T	0.774^*^	1.000										
SWC	0.718^*^	0.312	1.000									
pH	−0.791^*^	−0.756^*^	−0.184	1.000								
TN	0.403	0.565	−0.042	−0.519	1.000							
TP	0.788^*^	0.527	0.381	−0.862^**^	0.284	1.000						
AN	0.611	0.849^*^	0.021	−0.694	0.621	0.382	1.000					
AP	0.693	0.429	0.339	−0.786^*^	0.409	0.946^**^	0.295	1.000				
MBC	0.736^*^	0.545	0.326	−0.879^**^	0.344	0.934^**^	0.361	0.944^**^	1.000			
SOM	0.863^**^	0.839^**^	0.533	−0.708^*^	0.503	0.485	0.742^*^	0.345	0.446	1.000		
RM	0.867^**^	0.731^*^	0.376	−0.844^**^	0.549	0.924^**^	0.579	0.862^**^	0.883^**^	0.726^*^	1.000	
FY	0.874^**^	0.661	0.776^*^	−0.715^*^	0.429	0.762^*^	0.502	0.738^*^	0.732^*^	0.785^*^	0.821^*^	1.000

RS, soil respiration; T, ST at 10 cm soil (depth); SWC, soil moisture at 10 cm soil depth; TN, total nitrogen; TP, total available phosphorus; AN, available nitrogen; AP, available phosphorus; MBC, microbial biomass carbon; SOM, soil organic matter; RM, root biomass; FY, forage yield. * indicates *P* < 0.05, ** indicates *P* < 0.01.

## 4 Discussions

### 4.1 Different roles of N and P in affecting soil properties, root biomass, and alfalfa forage yield

In this investigation, soil temperature (ST) exhibited substantial variation in accordance with seasonal trends. The average ST demonstrated no significant difference among the CK, N, P, and NP treatments (**Figure 2**). The findings align with prior research (Liu et al., 2019; Sun et al., 2018; Wang et al., 2016). Likewise, no substantial variation in soil moisture was observed across the four treatments (**Figure 1**). Soil pH is a crucial fundamental physical and chemical characteristic of soil, serving as a significant indicator of acidity and alkalinity, which can influence nutrient availability for plants and microbial activity in the soil (Slessarev et al., 2016; Wagner et al., 2017). Certain research has indicated that prolonged fertilizing may lead to soil acidity (Ge et al., 2018; Schroder et al., 2011). In our trials, soil pH exhibited a decline in N, P, and NP treatments over two years, with the most pronounced decrease observed in the NP treatment. However, no significant differences were noted among the various fertilization treatments. This finding aligns with previous investigations in lowland tropical forest soils (Nottingham et al., 2015) and temperate forests (Zeng et al., 2018). Multiple studies have shown that the prolonged use of organic or inorganic fertilizers can increase soil organic matter (SOM) and total nitrogen (TN) concentrations through fertilizer immobilization and the enhancement of root biomass and residues (Bradford et al., 2008; Ge et al., 2018). This study found that P and NP treatments elevated the concentrations of SOM and TN. Simultaneously, N treatment elevated the soil’s TN content; however, it exerted little effect on the soil’s organic matter. This outcome may be attributed to the association of SOM with soil types, ecosystem types, soil horizons, forms of N and P, and fertilization timing (Cusack et al., 2010; Chen et al., 2017). Moreover, numerous studies have demonstrated that the impacts of N and P fertilization on soil microbial biomass carbon can be intricate. N fertilization produced varying effects on MBC, ranging from promotion (Wang et al., 2009) to inhibition (Li et al., 2010) or negligible effects (Wang et al., 2015). Compared with CK, N fertilization showed no significant effect on MBC in 2017 and 2018 (*P*>0.05), but phosphorus application significantly increased MBC in 2017 and 2018 (*P<*0.05). N and NP treatments exhibited no impact on MBC relative to CK in 2018(**Table 1**); The possible cause is that P addition may enhance microbial biomass; however, the suppressed microbial activity and diversity caused by N addition may obscure the effects of P addition on soil microorganisms when N and P are added simultaneously (Liu et al., 2019).

Fertilization with N or P is recognized for enhancing soil nutrients and alfalfa forage output in locations with ample rainfall and irrigation (Berg et al., 2007; Fan et al., 2016). This study revealed that N and P treatments enhanced forage yield, with the NP treatment resulting in a considerably higher yield compared to the control and the individual N or P treatments. The increase in plant height and leaf area resulting from N and P fertilization in agricultural soil may enhance solar radiation capture, improve photosynthesis, and ultimately lead to higher yields (Berg et al., 2007; Fan et al., 2016). Furthermore, the fodder yield markedly improved with elevated levels of soil AN and AP, indicating a co-limitation by these two nutrients on the productivity of the alfalfa pasture in this area (Jia et al., 2007; Li et al., 2018). Prior studies have demonstrated that optimal N or P fertilization can enhance root development (Fan et al., 2016; Li et al., 2018). The root system, serving as a primary organ for water and nutrient absorption and facilitating the exchange of substances and information between aerial and subterranean parts, significantly impacts the growth and yield of alfalfa (Fan et al., 2016; Xiao et al., 2015). The findings from this study indicated that root biomass was strongly influenced by N, P, and NP treatments over the two years. Nonetheless, no substantial changes were observed across the different fertilization methods. Several soil environmental parameters, including pH, organic matter content, temperature, moisture levels, and metal mobilization, significantly influence plant root development. The literature indicates that soil nutrients can markedly enhance crop root length density, root volume, root dry weight, and root penetration depth (Malhi and Gill, 2002; Yang et al., 2004). Our findings indicate that N and P fertilizers enhanced fodder productivity and root biomass primarily via modifying the physical and chemical features of the soil in this alfalfa grassland.

### 4.2 Effect of N and P fertilization and their interaction on the RS

#### 4.2.1 Individual effects of N and P fertilization on the RS

In the present study, alfalfa exhibited a relatively higher soil respiration rate compared with several annual crops and grasses (Wang et al., 2018a; Zhang et al., 2007). This may be attributed to its greater root biomass, sustained root exudation, and dense rhizosphere environment (Bedada et al., 2016; Li et al., 2018). Moreover, its soil respiration showed a more pronounced response to nitrogen and phosphorus fertilization. Additionally, compared with unfertilized natural grassland, the application of N and P fertilizers in this study significantly accelerated soil carbon emissions. These differences indicate that perennial forage crops such as alfalfa possess distinct carbon emission characteristics within agroecosystems, and greater attention should be paid to their role in regional carbon budgets.

This investigation revealed that N fertilizer led to increased respiration rates during the growing season and throughout the entire year (**Table 2**). This finding aligns with previous research indicating that N addition typically enhances root respiration in grassland ecosystems (Jia et al., 2012; Luo et al., 2016). N addition could stimulate RS by promoting plant growth and ecosystem productivity, which increases the availability of carbon substrates and further stimulates root and microbial metabolic activity, especially in grasslands limited by nitrogen (Fang et al., 2017; Jia et al., 2012; Luo et al., 2016). In the present study, N application alone significantly increased forage yield and root biomass compared with the control during the growing season, but showed no significant effect on soil microbial biomass carbon (*P*>0.05) (**Table 1**). In addition, soil respiration under nitrogen treatment was higher than that of the control. Moreover, no substantial difference was observed between CK and N therapy during the non-growing season. The findings are consistent with prior research (Fang et al., 2017; Luo et al., 2016; Zheng et al., 2018). This is likely due to soil temperatures remaining below 0 °C for the majority of the non-growing season, which can inhibit soil microbial and root activity, thereby diminishing the impact of N fertilizer on respiration (Han et al., 2018; Luo et al., 2016).

Research examining the impact of P fertilizer on RS is limited. This investigation revealed a beneficial effect during the growth season, with no effect observed in the non-growing season (**Table 2**). This disparity may be elucidated by variations in soil temperature, vegetative growth, and microbial activity (Fang et al., 2017; Feng and Zhu, 2019). In our study, P fertilization enhanced soil available P, root biomass, and microbial biomass carbon during the growing season. This finding corroborates multiple studies indicating that P fertilization can directly enhance soil available P, thereby fostering plant growth. The resultant increase in plant biomass contributes additional carbon inputs to the soil through litterfall and turnover, subsequently stimulating microbial activity and, consequently, stimulating soil respiration (Fang et al., 2017; Feng and Zhu, 2019).

#### 4.2.2 Combined and interactive effects of N and P fertilization on the RS

The repeated-measures ANOVA test in this investigation revealed no significant interaction effects of N and P fertilization on the RS (**Table 3**, *P* > 0.05), corroborating the findings of Ren et al. (2016). Liu et al. (2019) demonstrated that N and P inputs interactively affected root respiration in a subtropical forest in China. The interaction effects of N and P fertilization on root systems are inconsistent and contentious across many terrestrial ecosystems due to variations in fertilization rates, vegetation types, and measuring methodologies (Fu et al., 2013; Liu et al., 2019). Compared with sole N or P addition, combined NP fertilization increased soil available P (AP) and promoted root growth, as indicated by larger root surface area and longer root length. These changes in root morphology typically stimulate root respiration (Malhi and Gill, 2002; Wang et al., 2016). In addition, some studies have suggested that NP application reduces soil pH, which is widely considered inhibitory to soil microbial communities. (Van den Berg et al., 2005; Tian et al., 2016). In the present study, however, microbial biomass was enhanced under NP treatment even with decreased soil pH. This response may be explained by greater rhizodeposition and increased labile carbon inputs associated with improved root growth (Nottingham et al., 2015; Ren et al., 2016). Both increased root growth and higher microbial biomass would theoretically promote soil respiration (RS). Yet no significant differences in RS were detected among N, P, and NP treatments (**Table 2**, *P*>0.05). We suggest that the stimulatory effects of greater root and microbial biomass on soil respiration were offset by lower microbial specific activity and reduced root exudation under acidification, resulting in statistically similar soil respiration across treatments (Chen et al., 2016; Liu et al., 2019).

### 4.3 Effect of N and P fertilization on *Q*_10_

Our study revealed a mean *Q*_10_ value of 3.75 for the entire year, with a range of 3.13 to 4.22, surpassing the global average of 2.4 (Raich and Schlesinger, 1992) and the mean of 2.5 observed in a semiarid alfalfa pasture on the Loess Plateau (Fang et al., 2017). However, it falls within the range of 3.67 to 4.22 documented in previous research conducted in a meadow steppe of the Songnen Plain, China (Wang et al., 2018a). This suggests that the respiration of both microbial communities and plant roots at our study sites was more susceptible to fluctuations in soil temperature (Chen et al., 2010). The *Q*_10_ value is anticipated to remain constant over the course of a year when estimating annual soil carbon budgets. Nonetheless, mounting data suggest that the *Q*_10_ values of the RS rates fluctuate throughout the year (Han and Jin, 2018). In our study, *Q*_10_ was markedly elevated during the non-growing season (November to March of the subsequent year) compared to the growing season (April to October). This aligns with other prior research indicating that *Q*_10_ exhibits significant seasonal change in grasslands (Pang et al., 2013). The distinct variations in *Q*_10_ values throughout seasons may be ascribed to differing environmental variables, including the stability of soil organic matter, the physiology of soil microbes, root biomass, and substrate quality (Han et al., 2018). For instance, specific research indicates that *Q*_10_ diminishes with rising temperatures, while it also declines with reduced soil moisture (Han and Jin, 2018).

Multiple manipulation studies conducted over the last two decades have demonstrated that the impact of N and/or P fertilization on *Q*_10_ levels can be complex. Guo et al. (2017) indicated that prolonged nitrogen (N) and phosphorus (P) inputs diminish soil microbial respiration while increasing its temperature sensitivity in a Tibetan alpine meadow. Sun et al. (2018) indicated that the *Q*_10_ following NP fertilization diminished by 6.9% in low fertility soil, while remaining unaltered in high fertility soil in the Chinese Loess Plateau. Our work demonstrated an increase in Q10 value under nitrogen, P, and NP treatments, corroborating prior research indicating that N and P fertilization enhances the sensitivity of respiration rate to temperature fluctuations (Cusack et al., 2010; Pang et al., 2013). This suggests that soil nutrient availability may influence the Q10 value by altering substrate stability and the growth of above-ground and root biomass, thereby modifying the rate of carbon decomposition, which is likely to impact the *Q*_10_ value (Luo et al., 2016; Wang et al., 2018b).

## 5 Conclusion

This study examined the impacts of N, P, and NP fertilization on respiration rate (RS) and temperature sensitivity (*Q*_10_) through a field manipulation experiment. The outcomes of the current study indicated that nitrogen, P, and NP fertilization can lead to an increase in the respiration rate and temperature sensitivity of respiration. No interaction effects of N and P fertilization were detected. Moreover, *Q*_10_ was markedly elevated in the non-growing season compared to the growth season. RS has increased exponentially with ST at a depth of 10 cm and has shown a linear relationship with soil moisture, forage yield, and root biomass. Furthermore, RS exhibited a positive and significant association with total phosphorus (TP), microbial biomass carbon (MBC), and soil organic matter (SOM). Nevertheless, it exhibits a strong negative correlation with pH. These data demonstrate the response of the RS to nitrogen and P fertilization and their interactions. It is essential to consider the impact of fertilization regimes on *Q*_10_ when evaluating CO_2_ feedback to agroecosystems under future climate scenarios.

## Data Availability

The original contributions presented in the study are included in the article/supplementary material. Further inquiries can be directed to the corresponding authors.
